# Field Guide to Traction Force Microscopy

**DOI:** 10.1007/s12195-024-00801-6

**Published:** 2024-04-23

**Authors:** Aleksandra K. Denisin, Honesty Kim, Ingmar H. Riedel-Kruse, Beth L. Pruitt

**Affiliations:** 1 Department of Bioengineering, Stanford University, Stanford, CA 94305 USA; 2 Present Address: The Department of Pharmaceutical Chemistry, University of California, San Francisco, CA 94158 USA; 3 Department of Molecular and Cellular Biology, and (by courtesy) Departments of Biomedical Engineering, Applied Mathematics, and Physics, University of Arizona, Tucson, AZ 85721 USA; 4 Departments of Bioengineering and Mechanical Engineering, University of California Santa Barbara, Santa Barbara, CA 93106 USA

**Keywords:** Traction force microscopy, Cell biomechanics, Mechanobiology

## Abstract

**Introduction:**

Traction force microscopy (TFM) is a widely used technique to measure cell contractility on compliant substrates that mimic the stiffness of human tissues. For every step in a TFM workflow, users make choices which impact the quantitative results, yet many times the rationales and consequences for making these decisions are unclear. We have found few papers which show the complete experimental and mathematical steps of TFM, thus obfuscating the full effects of these decisions on the final output.

**Methods:**

Therefore, we present this “Field Guide” with the goal to explain the mathematical basis of common TFM methods to practitioners in an accessible way. We specifically focus on how errors propagate in TFM workflows given specific experimental design and analytical choices.

**Results:**

We cover important assumptions and considerations in TFM substrate manufacturing, substrate mechanical properties, imaging techniques, image processing methods, approaches and parameters used in calculating traction stress, and data-reporting strategies.

**Conclusions:**

By presenting a conceptual review and analysis of TFM-focused research articles published over the last two decades, we provide researchers in the field with a better understanding of their options to make more informed choices when creating TFM workflows depending on the type of cell being studied. With this review, we aim to empower experimentalists to quantify cell contractility with confidence.

**Supplementary Information:**

The online version contains supplementary material available at 10.1007/s12195-024-00801-6.

## Introduction

Traction force microscopy (TFM), first proposed in 1996 by Dembo et al. [1–3], enables quantitative measurements of cellular contractility as cells deform a compliant, linearly elastic substrate. The traction stress field is inferred from observations of cell-generated surface displacements by using a mechanical model of the material [4]. TFM has been used to understand the biomechanical factors impacting a range of biological processes, including stem-cell differentiation [5–7], maturation of stem cell-derived cardiomyocytes [8], and tumor cell force generation and migration [9–11].

Several review papers and tutorials exist to aid laboratories in setting up their own TFM experiments [4, 12–14], yet we have not found resources discussing TFM data processing steps and how experimental design choices contribute to sources of error within TFM workflows. Therefore, we will first focus on explaining the mathematical basis of common TFM methods and then move on to building an understanding of the assumptions of the mathematical methods and how experimental design choices lead to error propagation in the TFM workflow.

As an overview, in the following sections we will discuss TFM techniques (“TFM Techniques” section), present sources of error in TFM experiments based on experimental design and analytical choices (“Sources of Error in TFM” section), and provide discussion on strategies in TFM data processing (“Strategies in TFM Data Processing” section). Objectively selecting TFM analysis parameters for cells with different properties, particularly with low signal-to-noise ratio, can be challenging so we delve deeper into specific approaches used in the literature to tune these parameters in the “Choosing TFM Parameters for Challenging Applications” section. The “Matching Types of TFM Data to Analysis Pipelines” section focuses on helping TFM practitioners match their experiments to analysis pipelines. To further aid experimentalists, we analyzed 63 TFM-focused research articles from the last two decades to provide an overview of which TFM methods were used, how TFM data were reported, and the cell type analyzed with resulting maximum traction stress (Supplementary Table 1). In “Matching Types of TFM Data to Analysis Pipelines” section, we draw from our analysis of the literature and discuss ways to represent TFM data.

## TFM Techniques

Here we outline the basics of TFM. Other reviews of TFM provide in-depth details [4, 12, 13], and thus we focus on specifics crucial to regularization parameters used in the calculation of traction stress.

Briefly, the TFM workflow involves obtaining images of the surface of a material deformed by a cell, interpolating the displacement field generated by the cell on that material, and calculating the traction stress vectors that most likely led to the observed displacement field [15]. Linear elasticity assumptions are important to all TFM methods. Polyacrylamide has been widely accepted as a linearly elastic material under a wide range of strains (up to 90%) [16, 17]. Further, polyacrylamide hydrogels can be tuned to a specific stiffness (0.2–150 kPa [18, 19]) and can be functionalized selectively with extracellular matrix proteins [20]. Alternative substrates include silicones such as polydimethoxysilane, CY52-276A/B, and gelatin. It is important to verify that the material used for TFM satisfies the linear elasticity assumption and this can depend on a variety of factors including specific formulation of the material and how it was functionalized with extracellular matrix (how cells are tethered to the material). Fluorescent nanobeads embedded in the polyacrylamide act as fiducial markers for tracking displacement of the material. The material displacement is typically measured by comparing two images: one image with the cell contracted and one reference image in which the cell is removed or force-production is inhibited. A notable alternative approach that permits measurement of traction force in-vivo utilizes regular bleaching patterns in a fluorescent basement membrane as fiducial markers to measure tractions cells on the surface [21]. However, in-vivo measurements are challenging and correspondingly Yamaguchi et al. restrict their interpretation to qualitatively observing that the tissues are pushing themselves forward instead of pulling, which even without quantitative forces is a compelling result [21]. For this review, we will focus on studies employing characterized materials and in vitro experiments. Simultaneous control of cell force generation and TFM readout can be performed using a variety of techniques including optogenetics as demonstrated by Valon et al. [22]. Performing FRET (Förster resonance energy transfer) and TFM experiments on cells under the same conditions enables correlations of molecular and cellular tractions, as demonstrated a variety of papers: [23–26]. In vivo techniques are emerging but involve significant characterization of the model system to yield traction stress readout.

These images can be compared using a variety of algorithms to generate displacement vectors. Broadly speaking, these approaches can be split into two categories based on whether (1) regions or (2) discrete particles are tracked. In Particle Image Velocimetry (PIV) [27–30] and the related Digital Image Correlation [31], the image is split into regularly gridded regions. The regions from one image are then compared to slightly displaced regions in the second image using cross-correlation in an iterative process using progressively smaller window sizes to determine the most probable grid displacement. In optical flow [32, 33], the image once again is split into regularly gridded regions, but here the intensities between frames are subtracted and an advection equation is applied to describe the movement of the regions. Particle Tracking Velocimetry (PTV) [34] involves segmenting images to identify individual beads by locating the peak centroids and following the movement of these beads [35]. These techniques can also be used in combination. For example PTV can also be used after obtaining “coarse" information about displacements by correlation-based PIV [27, 36]. Once these displacements are inferred, a strain field is generated by assigning a vector from each position in the unstressed state towards the new position in the stressed state with a magnitude proportional to the displacement. The bead displacement tracking methods and substrate materials used by various groups are reported in Supplementary Table 1.

Conventional TFM with wide-field epifluorescence microscopy assumes that dominant stresses exist in the imaging plane and therefore neglects displacements in the out-of-plane axis. To gather information about cell traction forces in 3D, a confocal microscope must be used to measure vertical displacements [37, 38]. Digital volume correlation algorithms are needed to extend these 3D measurements in order to estimate traction stresses for cells embedded in 3D environments [37, 39, 40]. Note that in the Supplementary Table 1 “Traction Method” column we report whether the authors used 3D TFM. To be able to discuss the mathematics behind TFM in detail and point to potential sources of error, we focus on methods that measure bead displacements only in the plane of the substrate (2D). We will proceed to discuss underlying mathematical equations and mechanical assumptions below in the context of 2D TFM. Extensions to 3D can be made using similar sets of constitutive equations and regularization approaches, as well as alternative formulations. However, this extension is not trivial due to the added complexity of creating and solving for complex meshes in efficient and numerically stable ways (see below on FEM). We point readers to the following two manuscripts (Mulligan et al. [41], Feng et al. [42]) that describe applying the constitutive equations to 3D meshes.

Once the displacement field is obtained, TFM methods differ in how cell-generated traction stress is calculated. Direct TFM computes the spatial derivatives to the displacement field and uses constitutive relations to construct the strain tensor. The corresponding stress tensor is solved applying linear elasticity, equations of equilibrium, and appropriate boundary conditions. However, direct TFM is computationally expensive and noisy data can have a significant effect on the final result [43, 44]. In contrast, inverse TFM, an indirect TFM approach, solves an inverse problem of linear elasticity [4]. Inverse TFM uses elasticity theory to reconstruct the traction stress from displacement data. The Appendix contains more detailed mathematical background and assumptions to derive the inverse problem. Substrates used for TFM need to exhibit linear elasticity to calculate traction stress. If the stress–strain relation in a material is linear, it is linearly elastic and we emphasize that experimentalists should take care to validate this assumption for their particular formulation, thickness, functionalization, and other experimental parameters which may affect material properties.

Finite element discretization is a direct TFM method that estimates the force magnitude by discretizing the problem into smaller sections and solving a system of partial differential equations modeling elastic materials to minimize the distance between measured and computed displacements [4, 9, 38, 45, 46]. FEMs enable calculations of traction stress with no specific assumptions about the substrate [47] and can be applied to characterize 2D and 3D systems with nonlinear materials and arbitrary gel geometries [38]. FEM based approaches have relaxed the linearity assumption, for example Steinwachs et al. [48] provide an example of 3D TFM with hyperelastic FEM in which they fit parameters to their non-linear constitutive model from biomechanical measurements. In the “Traction Method” column of Supplementary Table 1, we indicate whether the authors used FEM.

Fourier transform traction cytometry (FTTC) is the most frequently used inverse TFM method (34 of 63 papers in Supplementary Table 1). One reason for the broad adoption of FTTC is that this method is much more computationally efficient, enabling quick processing of TFM data on a personal computer. FTTC based data-processing pipeline is now also easily accessible to the scientific community through ImageJ [28] and Matlab [13, 49] plug-ins that generate both displacement and traction stress fields. We point readers to work by Schwarz and Soiné for a table of many software tools developed for TFM [4] and the TFMLAB Matlab toolbox [50]. For a comparison between direct and FTTC approaches, see work by Blumberg and Schwarz [51]. Due to the popularity and accessibility of FTTC, we will focus this review more on the details relevant to FTTC.

### Constitutive Equations used in FTTC

The constitutive equations used in FTTC stem from assumptions of small strain of a linear elastic material (see Appendix A). Furthermore, we assume that the traction stresses are produced in-plane and that tractions in the normal direction are negligible [52, 53].

The displacement at position $\textbf{x}$ caused by a single point force at a position $\mathbf {x'}$ is given by Eq. 1 [54],

1 $$\begin{aligned} \textbf{u}_s\mathbf {(x)} = \mathbf {G(x-x')f(x')} \end{aligned}$$

where **u** is the displacement data (m), **f** are the traction forces (N), and $\textbf{G}$ is a Green’s function defined in further detail below (m/N). The subscript *s* indicates the displacement is due to a single point force. Throughout this paper, bold font indicates a vector or matrix.

However, usually there are multiple forces acting on the surface. The displacement at a given point is due to the net contributions of all the traction forces acting around that point and is therefore calculated with the convolution integral shown in Eq. 2,

2 $$\begin{aligned} \mathbf {u(x)} = \int \textbf{G}(\textbf{x}-\mathbf {x'})\mathbf {f(x')}d\mathbf {x'} \end{aligned}$$

where as before $\textbf{x}$ are the positions where displacements are being calculated and $\mathbf {x'}$ are the positions of traction and the variable of integration [55].

The assumption of small strain allows us to add traction forces from different sources at a given point [54, 56].

Specific solutions to the Green’s function are known for particular conditions. The Boussinesq solution is appropriate when the material can be approximated as a semi-infinite elastic half-space [4, 54, 57]. In experimental terms, this approximation is valid when the displacements made by cells on the substrate surface are much smaller than the size of the gel in all dimensions.

The Boussinesq solution to the Green’s function for displacement on a two-dimensional surface of a semi-infinite half-space is expressed in Eq. 3 [31, 36], where $\nu$ is the Poisson’s ratio and *E* is the Young’s modulus (N/m^2^) for the substrate.

3 $$\begin{aligned} \mathbf {G(x-x')} = \frac{1+\nu }{\pi E (\mathbf {x-x'})^3}\begin{bmatrix} (1-\nu )(\mathbf {x-x'})^2 + \nu x^2 &{} \nu xy \\ \nu xy &{} (1-\nu )(\mathbf {x-x'})^2 + \nu y^2 \end{bmatrix} \end{aligned}$$

The inverse problem approach involves solving for the traction stress with a system of linear equations using Green’s function to fit equilibrium solutions to measured displacements [4, 31]. The inverse method is applied in approaches such as the boundary element method [1, 58], but is computationally expensive [36]. FTTC addresses these issues by transforming the problem into Fourier space, removing the need for convolutions and reducing the problem to a simple matrix multiplication [59]. Equation 2 takes a simple form in Fourier space, i.e.,

4 $$\begin{aligned} \hat{\textbf{u}}(\textbf{k}) =\hat{\textbf{G}}(\textbf{k})\hat{\textbf{f}}(\textbf{k}) \end{aligned}$$

where carets indicate a Fourier transform and $\textbf{k}$ is the wave vector. $\hat{\textbf{f}}(\textbf{k})$ can now be solved for by multiplying both sides by $\hat{\textbf{G}}(\textbf{k})^{-1}$.

## Sources of Error in TFM

**Fig. 1:**
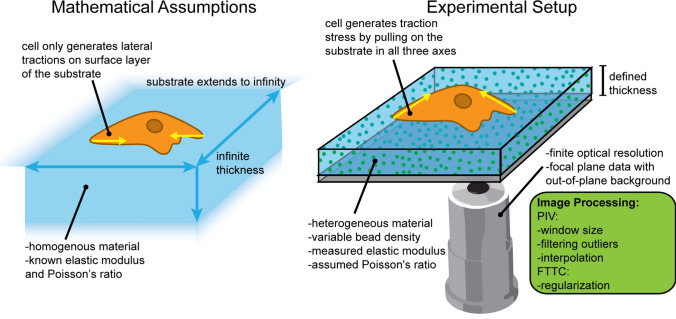
Sources of error in TFM processing steps stem from differences in the mathematical assumptions made in TFM calculations and the experimental setup. TFM calculations rely on key assumptions about a cell displacing just the top surface of a homogeneous, linearly elastic material with known elastic modulus and Poisson’s ratio. Experimental conditions differ from these mathematical assumptions, including: cell displacements on three axes, inhomogeneous materials with assumed mechanical properties, tracking displacement of the material using beads (which may be distributed unevenly), gathering out-of-plane displacements in addition to surface measurements, and image processing effects in both the PIV and FTTC steps

The inverse problem is ill-posed due to the continuous assumptions of the elasticity equations, which cause the calculated traction distributions to be very sensitive to noise in the displacement measurements [57]. Noise and systematic errors can be introduced and compounded through various steps in TFM experiments, from preparation of substrates to final force reconstruction (Fig. 1).

### Substrate Manufacturing and Mechanical Properties

Errors can impact the inferred traction stress starting from how the compliant cell culture substrates used in TFM are manufactured (Fig. 1). Incorrect values for the elastic modulus and Poisson’s ratio of the substrate introduce error into the Green’s function. While it may be convenient to use the elastic moduli from published formulations, variabilities in protocol can potentially lead to mechanical differences and therefore the mechanical properties of the substrate should be directly measured. When using polyacrylamide gels, significant material heterogeneity can also occur for certain formulations [60]. We strongly suggest that experimenters perform their own characterization of TFM substrates using a microindentation approach, such as Atomic Force Microscopy, to mimic mechanics at the cell-scale [61]. Mechanical characterization enables experimenters to derive specific elastic moduli for their materials and understand the error entering their TFM analysis from variability in mechanical properties when fabricating substrates. Differences in substrate stiffness can introduce a secondary experimental variability: cells tend to display higher spread area and force generation on stiffer substrates [62]. Therefore, experimenters should use caution when comparing cellular tractions under varying substrate stiffness and use proper controls.

The density of fluorescent beads in the substrate also impacts the resolution with which the displacement of the material can be calculated [12, 35, 63, 64]. When imaging the substrates, while higher bead density enables finer traction reconstructions, optical-resolution places an upper limit on how dense the beads can become before they can no longer be resolved from one another [35, 36, 63–66]. TFM experiments therefore often undersample the displacement field resulting in artificially coarse grain reconstruction of the traction forces [63, 64]. Various super-resolution techniques have been applied to recover these finer features [63, 64, 67, 68].

### Semi-infinite Half-Space Assumption

The Boussinesq solution (Eq. 3) requires assumptions of linear elasticity of the substrate and that the substrate can be treated as a semi-infinite half space. But, how does one ensure that the strains measured within the experiment satisfy these conditions? For polyacrylamide, the linear range for strain is up to 90% [16, 17] and therefore not a limiting factor. The semi-infinite half-space assumption turns out to be more restrictive. The assumption requires lateral strains to be small compared to the lateral dimensions and thickness of the gel. Consequently, many papers operate with the heuristic that displacements should be less than 1–5% of the gel thickness [39, 53, 58, 69]. However, this guideline is theoretically insufficient to guarantee the semi-infinite half-space assumption. In an early paper, Butler et al. note that in the extreme case where a focal adhesion covers the entire gel surface, even a small lateral displacement will simply shear the gel and therefore “experience” the lower fixed boundary condition [59]. While this scenario is unrealistic, del Alamo et al. used spectral analysis to determine the length scale of tractions that make up the solution and found the peak contributions corresponded to a cell length [69]. Alternatively, Sen et al. used finite element modeling to show that lateral and depth sensing of cells depends on their type, morphology (size and height), and gel stiffness [70]. In general, Sen et al. concluded that the depth-sensing length scale of most cells is 1–2 $\upmu$m (dominated by focal adhesions) and lateral-sensing decays within the first 2 $\upmu$m as well [70]. Altogether, it is advisable to ensure that cell-induced displacements are within low single-digit percentages of the gel thickness and to make sure the gel is thicker than the length scale of the cell. Unfortunately, using thicker gels can preclude the use of higher resolution objectives since gels are often imaged using an inverted microscope. Alternatively, computational approaches that avoid the infinite half-space assumption can be used to bypass these trade-offs [39, 53, 69].

### Out of Plane and Out of View Tensions

The presence of out-of-plane fluorescence from the beads embedded below the substrate surface also are significant sources of error. The Airy disk patterns of subsurface beads can be clearly visible in images taken with widefield fluorescence microscopy, which has the potential to complicate the tracking of the displacement field. Given an applied traction, beads on the surface will displace further than beads below the surface, with the displacements becoming vanishingly small far from the surface. The severity of this problem depends on the thickness of the optical slice taken. Furthermore, the analysis done by del Alamo et al. found that their semi-infinite half-space error increased with the height mismatch between the plane of the gel surface and the plane of the fiducial marker beads [69]. Therefore, to avoid out-of-focus fluorescence it is advisable to use a higher numerical aperture objective, or to use confocal microscopy [36, 71]. Another complementary approach is to use a protocol that ensures beads are only embedded on the surface of the gel during fabrication [63, 64] or by using a specific chemistry [72]. Restricting beads to the surface also avoids the potential error of focusing on the wrong plane. Altogether, the ideal experimental setup avoids out-of-plane signal through an optical setup with a thin focal plane and by restricting bead localization to the surface of gels.

The field of view must be carefully chosen because substrate displacements propagate beyond the boundary of the cell. Sufficient padding around the cell is necessary to capture all the relevant lateral displacements. Simultaneously, it is important to avoid cells outside the field of view, but near the boundaries since the displacements of these cells can propagate into the field of view. Tractions occurring outside of the field of view of a TFM analysis will contribute to the results physically but cannot be accounted for mathematically. These extraneous displacements not only contribute spurious tractions near the edges of the traction map, but also seriously compromise the zero-net force assumption in FTTC. Additionally, cells can influence the behavior of other proximal cells, complicating data analysis. For example, Tang et al. studied cardiomyocytes coupling across various separation distances and gel stiffness to derive a functional coupling that depends on the mechanical properties of the substrate and a cell-type specific constant [73]. Further, cell-cell spacing is often optimized for coupling if this is important for physiological function [47, 73], highlighting the importance of considering neighboring cells when evaluating single cells or cells on protein patterns. To determine the proper amount of padding required around the cells, the experimenter should measure displacements around a cell and determine the distance at which displacements are indistinguishable from background.

While the majority of cell tractions are in-plane with the substrate, there exists some out-of-plane displacement [38–40, 66] which can also contribute error to the calculation. This is especially true if the Poisson’s ratio of the gel is $<0.5$ which necessitates that the in-plane tractions are not independent of the out-of-plane component of the traction since the material is not perfectly isotropic [47]. Image-processing schemes for aligning images and calculating displacement fields, particularly window sizes for PIV-based image analysis [36, 74], can contribute significant errors to the TFM pipeline [35]. Rotations of the sample should be avoided as this can impede bead tracking algorithms. Filtering outliers by methods that detect and replace erroneous vectors with a median value from neighbors (the Normalized Median Test) may also contribute noise [75].

## Strategies in TFM Data Processing

**Fig. 2:**
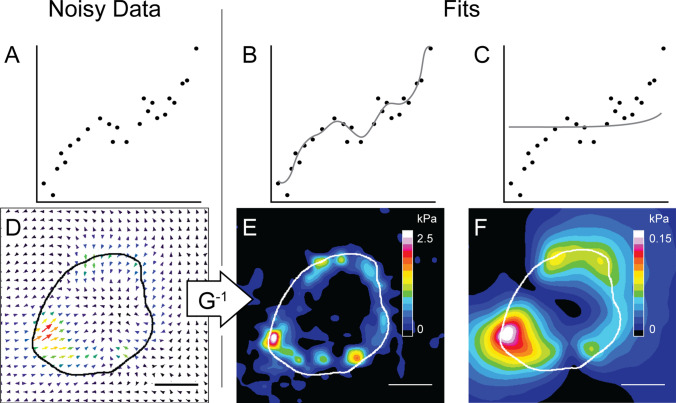
Regularization strongly affects solutions. **A** Noisy measurements (black dots) can be used to reconstruct the solution. **B** With little or no regularization, the solution tends to overfit. **C** When regularization is too high, the solution tends to underfit. **D** By analogy, bead displacements in TFM are noisy measurements of force. **E** Without regularization, the reconstructed force field is overfit to noisy contributions and tends to be overly discretized with high traction stress magnitudes. **F** Excessive regularization results in an over-smoothened reconstructed force field with low peak tractions spread over large areas. Scale bars in **D**–**F** represent 50 $\upmu$m

The many sources of error discussed so far results in data that is noisy and can produce erroneous traction fields using FTTC. Therefore, it is important to systematically limit the contributions of noise in the solution. A common and effective strategy for dealing with noisy data is to use regularization [36, 76]. In regularization, a term is introduced into the equation to penalize certain features. For example, consider the noisy data in Fig. 2A. By observation, it is obvious that a third-order polynomial would fit the data well. Without regularization, the solution will minimize the error between the data and the fit. While this solution will pass near all data points, it also produces excessively complicated solutions such as the higher-order polynomial in Fig. 2B. Therefore, introducing a regularization term that promotes simpler solutions by penalizing higher-order polynomials may be desirable. However, this strategy introduces yet another problem. If the regularization is too strong, then the solution may deviate greatly from a large number of data points in order to enforce a lower-order polynomial (Fig. 2C).

Similarly, in TFM, bead displacements should be viewed as noisy data (Fig. 2D). Without regularization, reconstructed forces tend to overfit the noise in the measurement, resulting in highly discretized and high peak magnitude tractions (Fig. 2E). Excessive regularization produces the opposite result: highly smoothed traction distributions with low magnitude (Fig. 2F). Note that the peak traction stresses for the cases with low and high regularization vary by several orders of magnitude.

### Tikhonov Regularization

The most popular implementation of regularization in TFM is zeroth-order Tikhonov Regularization [36, 36, 40, 49, 57, 65, 71, 77–80], which we introduce intuitively below. Zeroth-order Tikhonov Regularization takes the form shown in Eq. 5 [36].

5 $$\begin{aligned} \hat{\textbf{f}}_{\lambda } = \underset{\hat{f}}{\text{argmin}} (|| \hat{\textbf{G}}\hat{\textbf{f}} -\hat{\textbf{u}} ||^2_2 + \lambda ^2 || \textbf{L}\hat{\textbf{f}}||^2_2) \end{aligned}$$

where $\hat{{\textbf {f}}}$ is the computed traction field (N), $\hat{{\textbf {u}}}$ is the measured displacement field (m), $\hat{{\textbf {G}}}$ is the Green’s function (m/N), and $\lambda$ is the regularization constant (1/N). This equation returns $\hat{\textbf{f}}_\lambda$, which is the value of $\hat{\textbf{f}}$ for a given $\lambda$ such that the function inside the argmin() is minimized. $||\cdot||_2$ indicates a Euclidean or L2 norm. Note the arguments of the functions have been omitted for brevity.

The $|| \hat{\textbf{G}}\hat{\textbf{f}} - \hat{\textbf{u}} ||^2_2$ term is commonly referred to as the residual norm, because it measures the error between the measured displacements ($\hat{\textbf{u}}$) and the reconstructed displacements ($\hat{\textbf{G}}\hat{\textbf{f}}$). The $|| \mathbf {L \hat{f}}||^2_2$ term is referred to as the solution norm and measures the size of the solution $\hat{\textbf{f}}$ multiplied by some matrix $\textbf{L}$. Often, $\lambda$ is chosen by balancing the size of the residual norm versus the solution norm.

The $\textbf{L}$ matrix determines what features of the solution are weighted in the solution norm. The ‘zero’ in zeroth-order Tikhonov regularization stems from the specific choice of $\textbf{L}$ as the identity matrix, which like a zeroth-order derivative does not change the function. First- and second-order schemes employ a form of $\textbf{L}$ that approximates first- and second-order derivatives, but the zeroth-order scheme has been reported to be superior in TFM [36]. In practice, the zeroth-order scheme smooths out the reconstructed traction because high spatial frequency solutions (i.e. solutions with punctate and noisy stresses) tend to produce larger norms [36]. Note, the ‘L’ in L2 norm does not refer to the $\textbf{L}$ matrix.

Implementation of Tikhonov regularization results in the form shown in Eq. 6,

6 $$\begin{aligned} \hat{\textbf{f}}_\lambda = (\hat{\textbf{G}}^{T}\hat{\textbf{G}} +\lambda ^2 \hat{\textbf{H}})^{-1}\hat{\textbf{G}}\hat{\textbf{u}} \end{aligned}$$

where the carets denote a Fourier transform and $\hat{\textbf{H}}$ is the Fourier transform of $\textbf{L}$ squared.

The FTTC ImageJ plugin by Tseng implements this form of regularized TFM [76]. See Appendix A and papers by Sabass, Houben, et al. for further details about the derivation of this form [36, 81].

### Other Regularization Schemes

Tikhonov regularization uses the L2 or Euclidean norm, but this is not the only option. Several publications implemented the L1 or taxicab norm and reported superior performance over the L2 norm [52, 82, 83]. This improvement stems from the sparse nature of cellular tractions in the spatial domain. Sparse data consists mostly of zeros and cellular tractions are sparse because they are restricted to focal adhesions which are much smaller than the cell. Sparsity allows application of the signal processing technique known as compressed sensing which allows high-resolution reconstructions using a lower sampling rate than typically required [52, 82, 83]. However, L1 regularization is computationally expensive because cellular tractions are only sparse in the spatial domain, denying the advantage of efficient computations in Fourier space [52, 83]. If detecting weak signals such as nascent focal adhesions is critical to a project, then L1 regularization may be worth the computational cost.

Huang and co-authors implemented regularization strategies from other fields to TFM, which have the advantage of not requiring input of manual parameters [84]. In one approach, the authors split the regularization parameters into an $\alpha$ and $\beta$ term, where $\beta$ captures contributions of background noise, and employed Bayesian approaches in which new evidence is used to update an earlier model to calculate these parameters. While $\alpha$ and $\beta$ can be computed numerically, they also implemented a faster simplified scheme in which $\beta$ is calculated directly from displacement data far from the cell, thus simplifying the optimization to only the alpha parameter and yielding tractions which limit exceedingly high traction values and background noise. Huang and co-authors also implemented an Elastic Net regularization approach, a method from the computer vision field that combines L1 and L2 regularization, which yielded the most accurate traction reconstructions in the presence of noise for all the methods tested[84]. While these improvements on TFM processing are exciting, they may all still be too computationally expensive for practical use by many mechanobiology labs. The authors report the Bayesian L2 and Elastic Net methods take 45 to 360 times longer per image frame than the typical Tikhonov Regularization approach, respectively, and for their sample data it took several minutes to nearly an hour per data set [84]. However, Huang et al. also implemented a Fourier transform based Bayesian approach in a convenient Matlab package which they report to operate on the order of seconds [85].

To avoid ambiguity in the data processing methods and analyze dynamic processes (i.e., cell motility, cardiomyocyte contraction), some studies have opted to use raw substrate displacement values as a proxy for cell traction stress [86–89]. The downside to this approach is experimental results cannot be easily compared between different papers, as the raw displacements depend on substrate stiffness and other experimental conditions. In these cases, it is imperative that experimental conditions be well controlled and techniques to control cell shape and morphology such as protein patterning can help obtain reproducible results [86, 90]. Furthermore, some projects require insights into the actual forces exerted to make meaningful interpretations of the experiments. However, this alternative approach can useful if relative differences in contractility between experimental groups within one project is all that is needed.

## Choosing TFM Parameters for Challenging Applications

Choice of the regularization parameter $\lambda$ strongly influences the magnitude of the traction stresses inferred from displacement data and, if chosen poorly, could allow for amplification of background noise or underestimation of traction stress [52]. We thus looked at how the $\lambda$ parameter was chosen in various papers. We note that 15 [8–10, 12, 26, 36, 40, 49, 52, 57, 65, 71, 77, 80, 91] of the 21 papers that mentioned (or implied) use of zero-order Tikhonov regularization indicated the method by which the regularization factor $\lambda$ was determined. Some papers stated the $\lambda$ value used, but the authors did not explicitly explain how the value was chosen [78, 79, 92–95]. For investigations lacking information about the regularization factor, regularization may not have been used ($\lambda$ = 0). It is often difficult to determine how $\lambda$ was chosen because the $\lambda$ values and methodology for selection are often not reported in the literature.

Reporting the $\lambda$ parameter used in TFM analysis is important to understanding the degree of regularization applied to the data. We suggest that TFM users implement a reliable strategy to select the regularization parameter due to its extensive effect on traction-stress distributions, magnitude, and, ultimately, biological conclusions from the data [4]. We provide a rational approach to choosing a regularization parameter for TFM data in the following section.

### Using the L-curve to Minimize Solution and Residual Norms

**Fig. 3:**
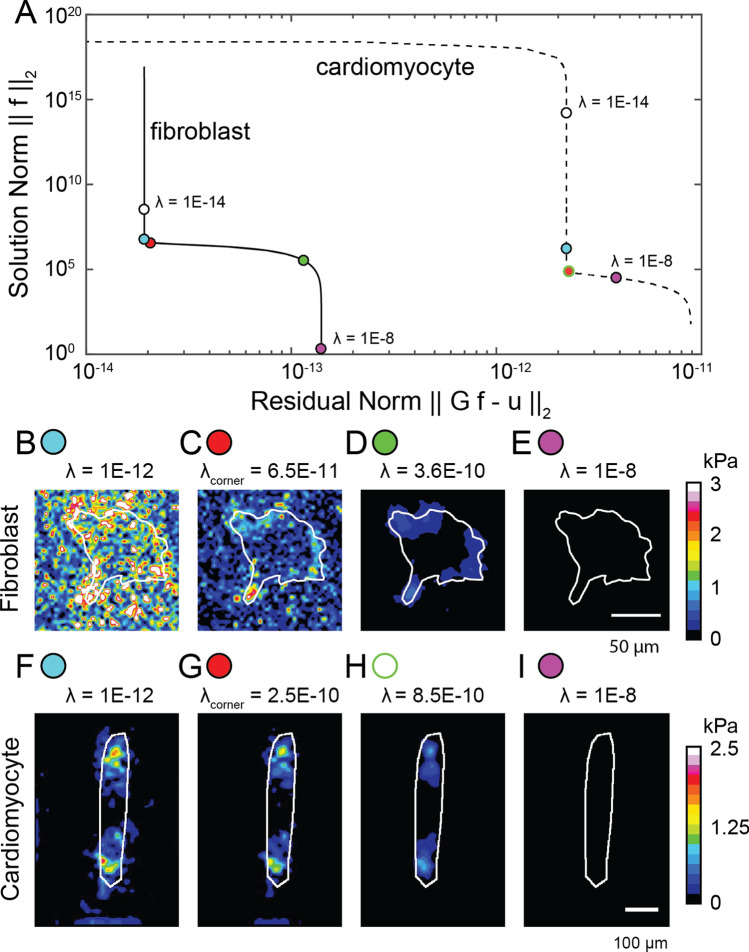
Regularization affects traction-force distributions and magnitude. **A** The L-curve shows the residual norm (x-axis) versus solution norm (y-axis) for a range of regularization parameters ($\lambda$) for a fibroblast and cardiomyocyte. **B**–**E** Tractions of a human foreskin fibroblast are discretized and high in magnitude with low $\lambda$. The distribution of tractions smoothens and traction magnitude decreases as $\lambda$ increases (**C** and **D**). **F**–**I** Tractions of a human cardiomyocyte differentiated from an induced pluripotent stem cell are less sensitive to changes in $\lambda$ than the fibroblast. White lines indicate the cell outline

A common approach to objectively selecting $\lambda$ is using the corner of the L-curve ( $\lambda _{\text{corner}}$ ) [57]. To generate the L-curve, the inverse problem is solved using a range of $\lambda$ values. Then, the residual ($|| \hat{\textbf{G}}\hat{\textbf{f}} - \hat{\textbf{u}} ||^2_2$) and solution ($|| \textbf{L}\hat{\textbf{f}}||^2_2$) norms are plotted (Fig. 3). The left side of the L-curve is populated by solutions that produce extremely high solution norms and the right side is populated by solutions that have large residual norms. The L-curve provides insight into the trade-off between the size of the regularized solution (solution norm) and the fit to the data given (residual norm) [96]. Finding the corner of the L-curve is an empirical method to balance this trade-off. Hansen created a useful, well-documented, and widely used MatLab package to locate the L-curve [96, 97]. We suggest that TFM users start their search for the optimal $\lambda$ for their experiment by evaluating the L-curves for a set of measurements.

### When the L-curve Fails

**Fig. 4:**
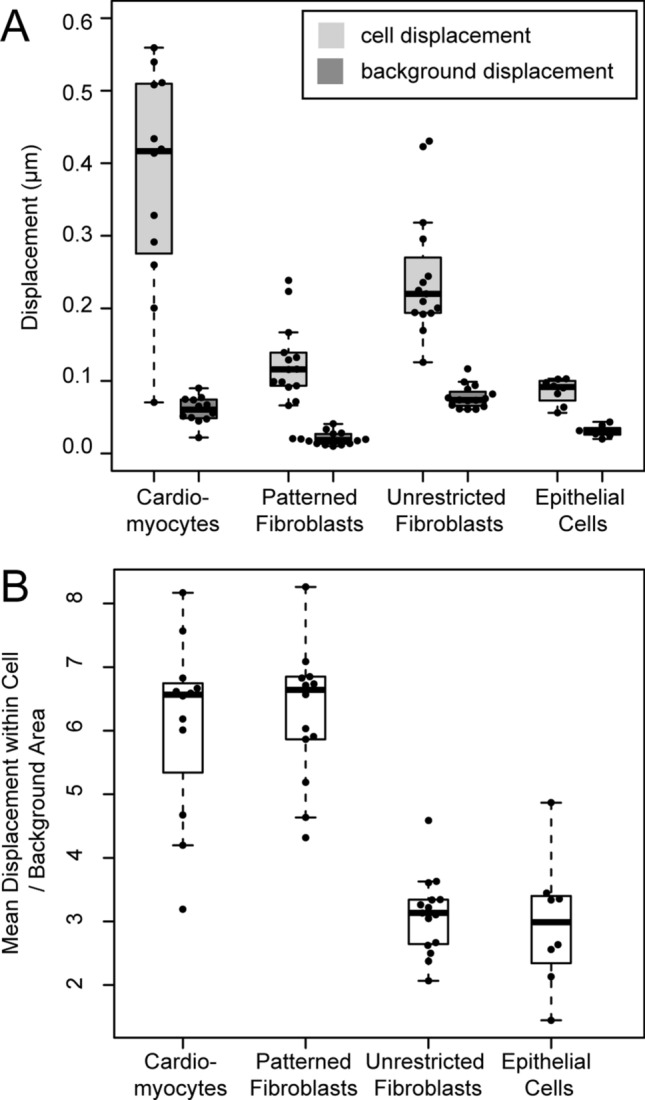
The signal-to-noise ratio for displacement is higher for cardiomyocytes and patterned fibroblasts compared to fibroblasts and epithelial cells. Signal is defined as the mean displacement within the cell boundary and noise is the displacement outside of the dilated cell boundary. Box plots are shown with whiskers extending to 1.5 times the interquartile range plus the third quartile (upper whisker) and subtracted from the first quartile (lower whisker)

While finding the $\lambda _{\text{corner}}$ minimizes the solution and residual norms (x-axis and y-axis of Fig. 3A–B), it may not be the best choice for all data and in some cases may not even be obvious to find. Figure 3 shows the L-curves and resulting traction stress maps for a human foreskin fibroblast and cardiomyocyte using different choices of $\lambda$ in the TFM analysis. Note the changes in the traction stress magnitude and distribution for the $\lambda _{\text{corner}}$ compared to other $\lambda$ choices for the fibroblast as compared to the cardiomyocyte sample. The signal-to-noise ratio of the data may impact the degree to which $\lambda$ contributes to changes in the magnitude and distribution of traction stress. For example, the $\lambda _{\text{corner}}$ may fall short whenever the signal-to-noise ratio is low, such as for fibroblasts or epithelial cells, while cardiomyocytes for which signal-to-noise ratio is higher may be less sensitive to how the regularization parameter impacts the output of TFM (see Fig. 4).

An important qualitative test is to verify that appreciable tractions do not occur far outside the footprint of the cell. Over-regularized solutions will artificially smear the results leading to large islands of traction that propagate far beyond the cell. Conversely, under-regularized solutions will lead to large punctate tractions that exist entirely outside of the cell boundary. In practice, the discrete nature of marker beads, as well as the presence of background noise means it is often impossible to neatly confine reconstructed forces within focal adhesions. While the threshold depends on experimental conditions, restricting tractions to the cell boundary dilated by $\sim$ 10% is a reasonable starting point. TFM users can analyze the traction stress map resulting from $\lambda _{\text{corner}}$ to evaluate background signal (where there is no adhered cell) and compare the cell-generated tractions to expected outcomes. We urge readers to develop a methodology to choosing $\lambda$ values in their TFM pipeline that is based on expected traction stress (using biophysical models and co-localization experiments for proteins that are known to be mechanically active).

As we evaluate the traction stress outside the cell boundary, we see that the $\lambda _{\text{corner}}$ choice for the fibroblast in Fig. 3 may not be appropriate for these data since we expect negligible traction stress in the “background” areas (see spatial patterns to signal outside of Fig. 3C–G and H–L). Although the traction-stress distribution within the cell area appears to follow the expected distribution of focal adhesions (by size and density), the solution is flawed because the traction stresses in areas outside of the cell are high. If $\lambda$ is too low, then the traction-stress solution is being fit to the noise rather than to cellular contractility. Tractions that extend beyond the cellular footprint, but have a significant portion directly underneath the cell, are acceptable (Fig. 3E, F). While the traction stresses in theory should not exist outside of the cellular footprint, in practice the limited spatial resolution of the reconstruction often generates tractions that are larger than focal adhesions and consequently extend beyond the cellular footprint [65].

We caution TFM users against comparing their measurements to cell tractions measured on different platforms, such as micropillar arrays [98, 99]. Micropillar arrays are free-standing microscale elastomeric features created in specific geometries that dictate the effective stiffness of each pillar. We refer readers to reviews by Schoen, Ribeiro, et al. for detailed discussion on how cell tractions on micropillar arrays compare to those on TFM on hydrogels [98, 99]. Briefly, although the effective stiffness of micropillar arrays and gels can be matched mathematically (see formulas reviewed by Schoen et al. [98]), cells may interact in a fundamentally different way with discrete micropillars than with continuous hydrogel substrates. We know of only one such comparison between the two substrate types, for E-cadherin coated micropillars and polyacrylamide gels [100]. Comparison to molecular-level measurement, via Förster resonance energy transfer tension sensors integrated into different load-bearing proteins, is also difficult. In this system, forces are inferred from the displacement of a calibrated molecular spring [101] and could yield confounding effects due to background signal and an unknown percentage of engaged proteins being analyzed [102]. Furthermore, Wu et al. used different methods for measuring cellular mechanics to reveal 100–1000-fold differences in the measured elastic properties of MCF-7 cancer cells because the measurement length scale and technique have a significant impact [103].

### Cell Type and Expected Displacements

We compared the signal-to-noise ratio in traction force data from several different experiments which use different cell types (Fig. 4). Some cell types (e.g. cardiomyocytes) produce much higher displacements than others (e.g. epithelial cells) (Fig. 4A). We measured the experimental signal-to-noise as the mean displacement of the substrate within a cell boundary over the displacement in the background. High displacements produce better signal-to-noise ratio (Fig. 4B). However, we found that signal-to-noise could vary significantly even for the same cell type when comparing data from two different experimenters using fibroblasts (Fig. 4A, B). Data with high signal-to-noise ratio works well with the L-curve, but data with low signal-to-noise ratio is under regularized and produces erratic tractions with the L-curve. Therefore, when the L-curve fails the first step is to consider modifications to the protocol to minimize the background noise of the experiment. In particular, rejecting out of plane signal by using a higher NA objective or confocal microscopy and taking steps to minimize disturbing the sample between acquisitions are good first steps (see above section on Sources of Errors in TFM). However, in some cases the cells produce low forces and even with low noise the signal-to-noise ratio remains poor, or other practical experimental considerations prohibit strategies for lowering the background noise.

One alternate approach to finding the appropriate $\lambda$ value is to use the Discrepancy Theorem, or $\chi$-criterion, which involves choosing $\lambda$ based on an expected value for the residual norm [57]. The Discrepancy Theorem posits that the optimal fit for the solution has a specific residual norm that depends on the standard deviation of noise in the experimental data and the expected number of data points [4]. Essentially, the estimated noise of the system sets a limit for how well the solution can fit the data by suggesting a specific value for the residual norm [56]. Reconstruction of the solution is limited by the noise; the Discrepancy Theorem limits the solution space because only a specific residual norm (the difference between the applied solution and the measured displacements) can be achieved. When noise makes a considerable contribution to the data, the $\lambda$ associated at the $\lambda _{\text{corner}}$ would have a residual norm much lower than that specified by the Discrepancy Theorem (see Appendix B).

Due to the significant role of the regularization parameter in TFM outcomes, Soiné et al. suggest applying a biophysical model to eliminate the need for regularization by restricting possible traction stress solutions based on biophysical limits [104]. Appropriate biophysical models require visualization of stress fibers and focal adhesions, which may not be available for every situation.

### Global vs Local $\lambda$

Once a regularization parameter has been found that produces quality data, one last issue remains. The optimal regularization parameter will vary for each traction map. As demonstrated, reconstructed traction maps are sensitive to regularization parameter values. Therefore, using different optimal values for each sample introduces the risk that differences in measurements between samples artificially arise from the regularization parameter. This makes it unclear whether the correct approach is to use a single regularization parameter for a given set of data, or to use the optimal value for each image.

Plotnikov et al. argue that once an optimal regularization parameter is found, it should be kept constant for the same cell type cultured on the substrates with a specific stiffness [12]. This requires the selection of some average $\lambda$ value. A reasonable approach is to find the optimal regularization parameter values from a random sample of images and use the mean value. However, different regularization parameters may need to be set between experimental conditions if cells have significantly differing mechanical properties, which reintroduces the uncertainty when ultimately comparing between experimental conditions.

On the other hand, using simulated data Kulkarni et al. found that individually optimizing regularization parameters produce more accurate traction reconstructions [105]. These results suggest that using the optimal regularization parameter for each image will most accurately reconstruct the cellular forces in real experiments.

Currently, the convention in the field is to use a global regularization parameter. We recommend this as the default option, but to keep in mind the alternative. Due to the sensitivity of traction magnitudes to the regularization parameter value, we recommend caution with either approach. In both cases it is important to try to keep experimental conditions such as frame size and bead density consistent, since these changes can affect the calculation of the optimal regularization parameter. Furthermore, wildly varying regularization values should be viewed with a critical eye. Finally, the paper should report whether a single average regularization parameter was used, or whether the parameter was optimized for each image.

## Matching Types of TFM Data to Analysis Pipelines

**Fig. 5:**
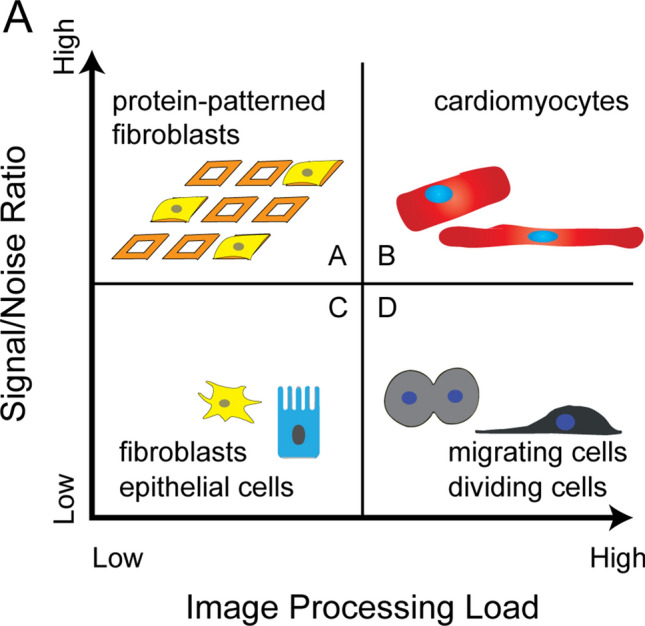
Experimenters should match TFM data processing methods based on the signal-to-noise ratio and image processing load. Broad classifications of TFM experiments fall into 4 categories: **A** cells patterned on proteins result in high signal and low image processing load, **B** cardiomyocytes which generate high forces and require analysis of multiple dynamic frames compared to reference, **C** epithelial and fibroblast cell lines on unpatterned protein generate low signal-to-noise ratio, and **D** similar cells which produce low traction forces and are dynamically migrating or dividing requiring comparisons of multiple frames against a reference

Broadly, we have found that TFM processing techniques can be categorized by two experimental parameters: image processing load and signal-to-noise ratio (Fig. 5). This produces four main groups which aid in selecting TFM data workflows. We defined image processing load as how many displaced bead images are compared to a reference to obtain PIV data. For some samples, one contracted image is compared to a reference while others (dynamic cells like cardiomyocytes) need to be evaluated over hundreds of frames to determine traction forces generated throughout the cell contraction cycle. Furthermore, higher-throughput assays demand a higher computational load [106]. Cells with high signal, regardless of image processing load, can be processed using FTTC and the L-curve criterion (Fig. 5, quadrants A and B). The regularization parameter for high image processing load samples (i.e., cardiomyocytes) can either be chosen per frame or set at a single value after deriving it from subset of the sample. Because FTTC is one of the fastest TFM processing methods, it is often the only practical solution for samples with high processing load.

For samples with low signal-to-noise ratio (Fig. 5, quadrants C and D), TFM workflow selection depends on image processing load. For low loads, using biophysical models [104], Discrepancy Theorem [4], or Bayesian frameworks [84] (discussed earlier) can yield accurate and robust data. Yet, these methods may be computationally prohibitive when moving towards larger image processing loads. With migrating or dividing cells, many frames need to be analyzed yet straightforward L-curve optimizations fail for identifying appropriate regularization parameters.

### Applying Machine Learning to Cellular Tractions

The advent of machine-learning tools offer a set of promising new approach to infer cellular traction forces. While an in-depth and comprehensive overview on this topic is out of the scope of this review, we provide the reader with some key points and references on where to read further.

One approach by Wang and Lin [107] trained a model to infer traction stresses from the displacement of fiducial beads without the need for apply methods like FTTC and the manual choice of regularization parameters. This model used a U-Net architecture and was trained on simulated data. They report that their model outperformed FTTC in inferring cellular tractions on their dataset. Furthermore, they report the model generalizes to a range of substrate stiffnesses and maximal tractions, and that the results obtained from experimental data qualitatively seem comparable to FTTC. Another approach by Li et al. [108] attempts to infer traction forces from wrinkles on thin films. Here, the authors use a combination of U-Net and generative adversarial networks (GAN) to extract features from image data and build a model for inferring tractions. Their model was trained on an experimental dataset that combined a thin deformable layer on top of a PDMS substrate with embedded fiducials. The fiducial layers were used to generate the traction fields for the ground truth. The authors report a good correlation in traction magnitude and direction. This approach potentially allows more freedom and ease in selecting and preparing substrates by removing the need to embed and track fiducial markers. However, it introduces a technical challenge of accurately detecting surface wrinkles. Another effort led by Schmitt et al. [109] demonstrates the inference of traction forces directly from fluorescent focal adhesion markers. Here the authors used a U-Net architecture trained on images of cells expressing various fluorescently tagged proteins on a polyacrylamide gel embedded with fiducial beads. Using the experimentally measured tractions as their ground truth, they found that the focal-adhesion protein zyxin in particular was sufficient to accurately reconstruct traction fields. The promise of this approach is that it removes the need for any special substrates, although it requires fluorescently tagged zyxin.

In general, machine learning approaches to TFM offer unique opportunities to improve data quality and offer greater experimental flexibility. However, care is needed to validate the models on any specific dataset, as the model may not generalize to all situations [110]. One solution is to apply transfer learning to further train models on the user’s specific experimental conditions, although this process may be a significant undertaking [111].

## Ways to Represent TFM Data

**Fig. 6:**
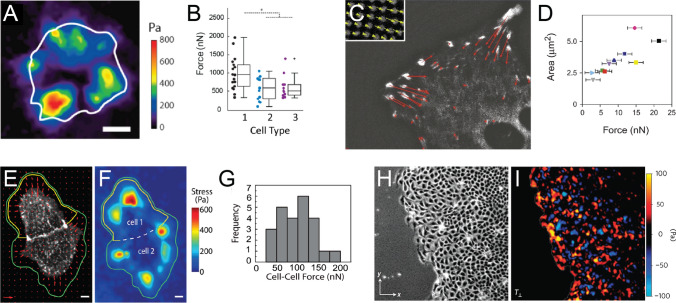
Several strategies can represent traction force data. **A** Most publications will include representative images of the generated traction maps and **B** quantification of tractions such as a total force per cell measurement. Fluorescently labelling cellular structures enables additional options for interpreting traction force data. **C** Labelling of focal adhesions enables constriction of traction forces to relevant sites and **D** additional insights such as the correlation of focal adhesion size and force. **E**–**G** Labelling of cell-cell boundary markers enables calculation of cell-cell forces through force balance requirements. **H**, **I** Traction force microscopy is not limited to single cells or small clusters of cells but can also be applied to larger units such as epithelial monolayers. **C**, **D** were adapted from [77], **E**–**G** were adapted from [120], and **H**, **I** were adapted from [136] with permission

Having determined how to optimally choose a $\lambda$ value, the next decision is how to best represent and analyze the data. We have tabulated the various methods for reporting data to assist the reader in choosing their methods (Supplementary Table 1). Here we offer a few suggestions previously reported in the literature and show vignettes from published studies as examples of TFM visualizations (Fig. 6).

The simplest representation is to reproduce the traction field maps and to qualitatively report the results (Fig. 6A). We found that 40 of the 63 papers (Supplementary Table 1) that we analyzed reported a traction magnitude map in their results section in order to depict the distribution and magnitude of cell traction stresses (Fig. 6B). Another approach is to report values such as peak or mean tractions [3, 26, 40, 112–114]. However, because tractions only occur at focal adhesion along the cell periphery, the data are dominated by low-magnitude background signal, which needs to be filtered when calculating the mean tractions. Unfortunately, there is no generally accepted way to distinguish background noise from signal. One strategy is to set a threshold for expected cell-traction forces and to define background signal as displacements that correspond to traction magnitude values below this threshold (see expected deflection calculation, Eq. 8). Another approach is to sum the magnitude of traction forces within the footprint of a cell [7, 8, 77, 78, 92, 115] or within a specific site corresponding to cell structures (such as focal adhesions, see Fig. 6C and D) [66]. It is also possible to calculate the strain energy (work per unit area) done by the cell on the material by integrating the product of traction stress and displacement [6, 59, 79, 93, 94, 116–118].

Visualization of focal adhesions through fluorescent markers enables a wider variety of options for analysis. Groups have reported the tractions per focal adhesion [57, 65, 77, 78, 119] as well as the angle between the principle axis of focal adhesions and the traction forces [40, 77, 80]. Further, visualization of focal adhesions provides a qualitative check to verify that tractions are localizing properly.

When analyzing multiple adhesive cells, TFM also affords the possibility of measuring the force between cells. Using force-balance principles, cell-cell tension between cell pairs can be measured (Fig. 6E-G) [94, 95, 120]. This type of analysis can be extended to any number of cells as long as they do not form a loop [94]. FEM based approaches can be used to calculate tension between cells in a cluster containing loops, such as stresses within a cell monolayer (Fig. 6H and I) [94, 121].

## Conclusions

TFM is a powerful technique to quantify cellular function by measuring how cells displace a compliant substrate. Laboratory tools to perform TFM experiments are easily acquired (polyacrylamide or other hydrogels, beads, functionalization chemistries) and researchers can quickly download various packages to perform TFM calculations [4]. With this review paper, we shared mathematical details of the most common TFM methods (PIV followed by FTTC). We have sought to make TFM more accessible by covering assumptions and considerations in the TFM workflow with highlights on how errors propagate to results and how to match TFM data processing methods to the type of experiment being performed. We hope that the strategies and background information outlined here will be helpful for researchers designing TFM experiments to better understand and characterize mechanobiology pathways.

### Supplementary Information

Below is the link to the electronic supplementary material.

Supplementary file 1 (pdf 178 KB)

## Data Availability

The raw data for Figures 3 and 4 are available from the authors upon reasonable request.

## Appendix A: Mathematical Background

Let us first consider continuum elasticity theory to understand the displacement of the soft elastic substrate by the cell. We can relate a strain tensor $\epsilon$ and a stress tensor $\sigma$ using material linearity assumptions when forces act on internal surfaces (full derivation in [4]), shown in Eq. 7,

7 $$\begin{aligned} \mathbf {\sigma } = \frac{E}{1 + \nu } \begin{bmatrix} \epsilon _{xx} + \frac{\nu }{1 - 2\nu } (\epsilon _{xx} + \epsilon _{yy}) &{} \epsilon _{xy} \\ \epsilon _{yx} &{} \epsilon _{yy} + \frac{\nu }{1 - 2\nu } (\epsilon _{xx} + \epsilon _{yy}) \end{bmatrix} \end{aligned}$$

where *E* is the Young’s modulus $\nu$ is the Poisson’s ratio of the substrate, and $\epsilon$ is the strain on the material, where the first subscript indicates the face acted on and the second subscript depicts the direction of the strain.

We can then extend the relationship to the displacement vector field and applied forces on the material using the Cauchy momentum equation,

8 $$\begin{aligned} \rho \frac{D\textbf{u}}{Dt} = \nabla \mathbf {\sigma } + \rho \textbf{g} \end{aligned}$$

which relates $\rho$ density of the continuum, ${\sigma }$ the stress tensor, **u** the flow velocity vector field dependent on time and space, and **g** the body forces per unit mass acting on the continuum (Eq. 8).

We can use conservation of linear momentum and simplify to get a balance of the internal and body forces on the continuum (Eq. 9).

9 $$\begin{aligned} \nabla \mathbf {\sigma } = - \rho \textbf{g} \end{aligned}$$

We can then combine Eqs. 7 and 9 [4] to yield Eq. 10.

10 $$\begin{aligned} \frac{E}{2(1+\nu )}\nabla ^{2}\textbf{u} +\frac{E}{2(1+\nu )(1-2\nu )}\nabla (\nabla \cdot \textbf{u}) = - \rho \textbf{g} \end{aligned}$$

Setting the body forces to zero because the displacements are due to forces on the surface [54], we can simplify Eq. 10 to Eq. 11.

11 $$\begin{aligned} (1-2\nu )\nabla ^{2} \textbf{u} +\nabla (\nabla \cdot \textbf{u}) =0 \end{aligned}$$

Following the derivation of Landau & Lifshitz (Section 8 in 2nd edition of *Theory of Elasticity*) [54], we arrive at the relationship for calculating the displacement caused at $\textbf{x}$ caused by a point force a distance $\mathbf {x'}$ shown in Eq. 12.

12 $$\begin{aligned} \mathbf {u(x)} = \mathbf {G(x-x')f(x')} \end{aligned}$$

where **u** is the displacement data, **f** are the traction forces, $\textbf{G}$ is a Green’s function defined in further detail below.

The Boussinesq solution to the Green’s function for displacement on a two-dimensional surface can be expressed as shown in Eq. 14 [31].

13 $$\begin{aligned} \mathbf {G(x-x')} = \frac{1+\nu }{\pi E (\mathbf {x-x'})^3}\begin{bmatrix} (1-\nu )(\mathbf {x-x'})^2 + \nu x^2 &{} \nu xy \\ \nu xy &{} (1-\nu )(\mathbf {x-x'})^2 + \nu y^2 \end{bmatrix} \end{aligned}$$

The displacement at a given point is due to the net contributions of all the traction forces and can therefore be calculated with the convolution integral shown in Eq. 13.  $\textbf{x}$ are the positions where displacements are being calculated and $\mathbf {x'}$ is the ‘dummy variable’ used in integration [55].

14 $$\begin{aligned} \mathbf {u(x)} = \int \mathbf {G(x-x')f(x')}d\mathbf {x'} \end{aligned}$$

Given the regularization scheme shown in Eq. 15.

15 $$\begin{aligned} \textbf{f}_{\lambda } = \underset{f}{\text{argmin}} (|| \mathbf {Mf -u} ||^2_2 + \lambda ^2 || \textbf{Lf}||^2_2) \end{aligned}$$

where **f** is the computed traction field (N), **u** is the measured displacement field (m), ${\textbf {M}}$ is a matrix derived from the Green’s function and numerical techniques (m/N), and $\lambda$ is the regularization constant (1/N). This equation returns $\textbf{f}_\lambda$, which is the value of $\textbf{f}$ for a given $\lambda$ such that the function inside the argmin() is minimized. $||\cdot||_2$ indicates a Euclidean or L2 norm.

However, calculation of ${\textbf {M}}$ is computationally expensive. Instead, it is much more efficient to convert take the Fourier transform of 13, which removes the convolutions from the equation as shown in Eq. 17.

16 $$\begin{aligned} \hat{\textbf{f}}(\textbf{k}) =\hat{\textbf{G}}^{-1}(\textbf{k}) \hat{\textbf{u}}(\textbf{k}) \end{aligned}$$

Using the same regularization scheme shown in Eq. 15,

17 $$\begin{aligned} \hat{\textbf{f}}_{\lambda } = \underset{\hat{f}}{\text{argmin}} (|| \hat{\textbf{G}}\hat{\textbf{f}} - \hat{\textbf{u}} ||^2_2 + \lambda ^2 || \hat{\textbf{H}}\hat{\textbf{f}}||^2_2) \end{aligned}$$

Minimization of the function occurs when the first derivative is equal to zero. After taking the derivative and setting equal to zero, we obtain Eq. 16 which can be solved for $\hat{\textbf{f}}$ [36] yielding the final Eq. 18.

18 $$\begin{aligned} \hat{\textbf{f}}_\lambda (\textbf{k})= (\hat{\textbf{G}}^{T}(\textbf{k})\hat{\textbf{G}}(\textbf{k}) +\lambda ^2 \hat{\textbf{H}}(\textbf{k}))^{-1}\hat{\textbf{G}}(\textbf{k})\hat{\textbf{u}}(\textbf{k}) \end{aligned}$$

## Appendix B: Discrepancy Theorem

Thus, the Discrepancy Theorem results in $\lambda$ values higher than ones suggested by the $\lambda _{\text{corner}}$ method. The residual norm for the optimal fit according to the Discrepancy Theorem is shown in Eq. 7,

19 $$\begin{aligned} || \mathbf {Mf - u} ||_2 = 2(N-M)\sigma ^{2} \end{aligned}$$

where *N* is the number of sites at which the traction stress is measured, *M* is the number of positions at which focal contacts are made between the cell and substrate, and $\sigma$ is the standard deviation of the distribution of measurement errors for the displacement vectors.

Equation 7 is based on assuming a Gaussian distribution for the noise in the displacement vector with standard deviation $\sigma$ [77]. By using the degrees of freedom (N–M) in the system [122], the Discrepancy Theorem allows for as much regularization as possible considering contributions from the noise to estimating the true traction-stress distribution.

To implement the Discrepancy Theorem, the user must make assumptions about the number of focal contacts in the sample and the standard deviation of the displacement vectors. One can estimate the number of focal contacts by visualizing focal adhesions or by estimating the percentage of the image area expected to be under cell traction. Since focal adhesions cannot be visualized in most cases, we choose to estimate the number of active cell tractions in each sample by evaluating how many displacement vectors achieve a magnitude greater than a specific threshold to qualify as a cell traction. We define the displacement threshold by setting a lower limit for the expected traction force and use linear elastic theory to calculate the expected displacement due to this traction force [123],

20 $$\begin{aligned} d = \frac{F(1+\nu )}{uE\pi } \end{aligned}$$

where *d* is the expected displacement in meters, *F* is the minimum traction force expected, *u* is the lateral resolution of the imaging platform used, and *E* is the elastic modulus of the substrate.

The expected force varies depending on the substrate stiffness and cell type used. We use Abbe’s diffraction limit for the lateral resolution, *u*,

21 $$\begin{aligned} u = \frac{\lambda _{emission}}{2NA} \end{aligned}$$

where *NA* is the Numerical Aperture of our objective and $\lambda _{emission}$ is the emission wavelength of our system.
