# Supplementary material for: Field Guide to Traction Force Microscopy
Source: Cell Mol Bioeng. 2024 Apr 23;17(2):87–106. doi: 10.1007/s12195-024-00801-6 (PMC11082129; doi:10.1007/s12195-024-00801-6)
Supplement: Supplementary file 1 — (pdf 178 KB) [file 12195_2024_801_MOESM1_ESM.pdf]

Appendix C. Supplementary Table 1: Summary of TFM Studies

Table C.1: Summary of TFM Studies

1st Author	Year	Cell Type	Sub Stiff (kPa)	Mat	Disp Method	Traction Method	Reg	Max Stress (kPa)	Data Reported	Ref
Dembo and Wang	1999	NIH 3T3	6.2	PA	bead registration and PTV	maximum likelihood using Boussinesq solution on mesh	increasing λ until χ^2 of particle displacement error reaches number of tracked markers	1-12.5	TM	[58]
Wang	2000	NIH 3T3	14, 33	PA	[58]	[58]		1.02 - 1.55	mean TF	[112]
Beningo	2001	Goldfish fin fibroblasts	24	PA	[58]	[58]	[58]	20	max FA TF/max cell TF	[119]

Munevar	2001	NIH 3T3	28	PA	CC	maximum likelihood Boussinesq solution on mesh		3	mean TF	[3]
Balaban	2001	HFF	12 - 1000	PDMS	WA	FTTC	ZO, used DT	5.5	TTF in FA, TF angle to FA	[77]
Balaban	2001	cardiac fibroblasts	12 - 1000	PDMS	WA	FTTC	ZO, used DT	5	TF versus FA area	[77]
Butler	2002	HASM	1.2	PA	normalized CC	con and unc FTTC		0.4	TM	[59]
Tolic-Norrelykke	2002	HASM	0.8 - 1.3	PA	CC with filtering	FTTC [59], con and unc		0.2	contractile moments, SE	[116]
Wang	2002	HASM	1.3	PA	CC	FTTC [59], con		0.08-0.45	TM, cell area versus TS	[117]
Wang	2002	HASM	0.87, 1.3	PA	CC [59]	FTTC con		0.9	contractile moment (SE)	[124]

Schwarz	2002	HFF	12 - 1000	PDMS	WA	SVD with regularization	ZO, used DT and LC	6.6E-7 N of force at FA	TM, TF magnitude	[57]
Marganski	2003	NIH 3T3	28	PA	optical flow algorithm	N/A - paper focuses on optical tracking	N/A	N/A	N/A	[125]
Marganski	2003	rat cardiac fibroblasts	9	PA	search for patch with similar intensity, interpolate	Bayesian likelihood function approximate deformation [1, 58]		3 - 6	TM	[126]
Curtze	2004	Ost	4.4, 6. 8	PA	CC optical flow [125, 58]	LIBTRC analysis		0.05	TM	[127]
Tolic-Norrelykke	2005	HASM	0.8 - 1.3	PA	image correlation [59, 116]	FTTC [59], con		0.325	TM, TF over time	[128]

Doyle and Lee	2005	fish kerato-cytes	?	gelatin	CC optical flow [125]	[58]		0.00013	TM, 90th % TS over time	[129]
Yang	2006	human tendon fibrob-last	3	PA	PTV, linear least squares fit	3D FEM, con		0.25	TM	[130]
Lombardi	2007	*D. dis-coideum*	2.5	gelatin	CC-based optical flow [58, 125]	LIBTRC analy-sis		1.3	TM, 90th % TS plotted with cell speed and shape	[131]
Chen	2007	rabbit fibrob-lasts*	3	PA	PTV	FTTC [59]		0.55	TM	[132]
Sabass	2008	MEF	15.6	PA	PIV and PTV	FTTC and BEM	ZO, LC	0.5 - 2.5	TM	[36]

Iwadate and Yumura	2008	*D. discoideum*	1, 2.8	CY52-276A/B silicone, gelatin	displacements of pre-defined nodes calculated from PTV	triangle FEM		1	TM, stress along cell edge over time	[133]
Iwadate and Yumura	2008	*D. discoideum*	1	CY52-276A/B silicone	displacements of pre-defined nodes calculated from PTV	triangle FEM		0.24	TM	[134]
Rosel	2008	neoplastic fibroblastoid LW13K2	13	PA	FTTC difference-with-interpolation image analysis	triangle FEM		0.5	TM, 50th % of TF generated along edge	[135]

Gardel	2008	PTK1	1.5 - 2.8	PA	CC-based tracking [36]	unc deconvolution [59]		0.1	TM	[36]
Huang	2009	NRVM	2	PA	DIC	modified Green's function to restrain noise		0.32	TM, max TS	[31]
Ambrosi	2009	T24	1.95, 6.3, 9.9	PA	PTV in ImageJ	numerical integration, adjoint equation based on minimization of coupled elliptic partial differential equations	LC	0.05 - 0.2	TM, velocity of migration, max TS/substrate stiffness	[9]
Trepat	2009	MDCK	1.3	PA	CC PIV	FTTC modified for finite thickness		0.1	TM, TS magnitude	[136]
Califano	2010	BAEC	1, 2.5, 5, 10	PA	CC-based optical flow	LIBTRC analysis		0.2 - 0.4	TS magnitude, TS/area	[62]

Jannat	2010	neutrophils from human blood	2, 12	PA	CC-based optical flow	LIBTRC analysis		1	TM	[137]
Stricker	2010	U2OS	2.8	PA	PIV software in Matlab (mpiv)	FTTC, TRPF	ZO for FTTC, LC for TRPF	0.09 - 0.150	TM, TS across FA	[65]
Legant	2010	BPASMC	0.5 - 2	PEGDA	nearest neighbors to create vectors	discretized Green's function	ZO with LC	2	TM, TS over time, mean TS at angle to cell center of mass	[40]
Tambe	2011	RPME, MDCK	1.3	PA	PIV	FEA		0.3	TM, normal stress	[121]
Prager-Khoutorsky	2011	HFF	4, 26	PA	[59]	FTTC [59]		0.5	contraction moment, SE	[138]
Betz	2011	NG108-15	0.11	PA	CC	unc deconvolution [59]		0.04	TM and internal stress maps	[139]

Maruthamuthu	2011	MDCK	8.4, 20.7	PA	PIV using Matlab, filtered, interpolated using kriging method	FTTC with regularization, FTTC without regularization, and BEM		0.5	TM, balance cell-cell and cell-ECM forces	[120]
Koch	2012	DRG	1	PA	CC	FTTC [36]		0.018 - 0.070	Peak, mean TS, noise TS over time	[113]
Marinkovic	2012	IMR-90	0.3, 1, 6, 13, 17, 20	PA	CC in Matlab [128]	con FTTC [59]		0.1 - 0.7	root mean squared TS	[140]
Oakes	2012	NIH 3T3	2.8, 8.6	PA	PIV-q	FTTC	ZO, unclear how λ is chosen [36, 65, 7]	0.7 - 2.2	FA TF, TTF	[78]
Oakes	2012	U2OS	2.8, 8.6	PA	PIV-q	FTTC	ZO, unclear how λ is chosen [36, 65, 7]	0.2	FA TF, TTF	[78]

Plotnikov	2012	MEF	8.6	PA	CC-based PTV	FTTC	2E-6, no justification for λ	2.7	TTF	[92]
Mertz	2012	mouse keratinocytes	3	CY52-276A/B silicone	centroid analysis,in Matlab	FTTC	λ = 9E-10, no justification	0.05	SE density, TS vectors	[93]
Tseng	2012	MCF10A	7	PA	PIV in ImageJ, normalized CC	FTTC with regularization scheme		0.5	TM	[28]
Hersch	2013	rat cardiomyocytes	15 - 90	PA	CC to template with sub-pixel accuracy [141]	superposition of point forces at cell adhesion sites [57, 142] and force field calculation [81]		0.08	TF sum over time	[115]
Peschetola	2013	T24, RT112	10	PA	PTV in ImageJ	FEM with bead positions	λ = 6E-7, chosen by LC	0.375	TM	[10]

Liu	2013	HeLa cells	2.5	PA	DIC	MATLAB program, specifics of process not specified		0.8	TM	[143]
Elkhatib	2014	NIH 3T3	5	PA	extract bead positions [139]	FTTC by Butler		0.3	SE	[118]
Ng	2014	MCF10A	8, 35	PA	CC	FTTC butler 2002, Sabass 2008	λ = 5E-8 to 1E-6, no justification	1	Force balance between cells, SE	[94]
Oakes	2014	NIH 3T3	16	PA	PIV in Matlab (mpiv)	FTTC	ZO, no justification for λ [36, 59]	2.5	SE, max TS	[79]
Plotnikov	2014	MDA-MB-231	8.6	PA	CC on smoothed data (median filtering)	FTTC	Bayes theory, calculate λ based on Gaussian distributions	0.8	TM	[12]

Bollmann	2015	microglial cells	0.1 - 1	PA	CC	FTTC		0.01 - 0.27	mean and peak TS	[114]
Han	2015	PTK1	8	PA	CC-based image tracking with sub-pixel registration	BEM	L1 and L2 regularization (Tikhonov)	3	TM, TS in nascent adhesions	[52]
Brask	2015	RPTP-α fibroblasts	8	PA	PIV (iterative window)	BEM with L1 reg, FTTC with L2	BEM - regularization not necessary. FTTC [144]	5	TM	[82]
Rosowski	2015	HESC	3	PDMS	centroid analysis in Matlab	FTTC		max SE: 2 pN/μm	SE density	[6]
Soine	2015	U2OS	8.4	PA	PIV-q in Matlab	FTTC	not needed, used biophysical models	12.6	TM	[104]

Ribeiro	2015	hiPSC-CM	10	PA	centroid analysis in Matlab	FTTC	λ = 9E-9, chosen by LC	0.016	TM, sum of forces, power	[8]
Sim	2015	MDCK	25	PA	PIV	FTTC	λ = 1E-9, no justification	0.255	TM, SE, cell-cell and cell-ECM force	[95]
Aratyn-Schaus	2016	miPSC-CM, mES-CM	13, 90	PA	PIV plug-in for ImageJ	FTTC		2	TTF	[7]
Bergert	2016	HeLa, REF, PC12 cells	1 to 20	CY52-276A/B	normalized CC	nonlinear framework based on boundary conditions, implicit FEA solver	no regularization required	12	TM	[46]

Chang	2016	HFF	18, 47	PA	PIV in ImageJ, normalized CC	FTTC	λ = 7E-10, 3E-10 chosen by DT	0.8	TM, mean TF	[26]
Sabass	2017	M. Xanthus	3.60E-04	silicone	CC-based tracking[36, 12]	FTTC	ZO, λ = 0.01 to minimize regularization	0.3	TM, local force in hotspots	[71]
Guo	2017	A7 cells, MEF, NIH 3T3, HeLa	2, 10	PA	image correlation method	FTTC [59]		0.7	TM	[5]
Sune-Aunon	2017	CHO-K1	5	PA	image registration then Otsu thresholding on displacement	FTTC	L1 regularization	2.9	TM	[83]

Ribeiro	2017	hiPSC-CM	10	PA	CC: DIC with Matlab Ncorr and PIV	FTTC	ZO, LC	0.6	TM	[49]
Collins	2017	MDCK	30, 60	PA	PIV in ImageJ, normalized CC	FTTC	λ = 1.3E-9, 6.6E-10 chosen by DT	0.05	TM, area of high stress regions	[91]
Makarchuk	2018	SW480	0.45	PA	CC based on intensity of particle center in 3D	applying Green's function	ZO, LC	1	normal TF, angle between major dipole axis of force field and cell axis	[80]
Lin	2018	MDA-MB-231	1, 5, 10, 20	PA	[31]	[31]		0.6	TM, SE	[145]

Abbreviations in heading: Sub Stiff - Substrate Stiffness, Mat - Material, Disp - Displacement, Reg - Regularization, Ref - Reference

Cell line abbreviations: NIH 3T3 - NIH 3T3 mouse embryo fibroblasts, HFF - human foreskin fibroblasts, HASM - human airway smooth muscle cells, PTK1 - female rat kangaroo kidney epithelial cells, T24 - human epithelial bladder cancer tumor cells, U2OS - human hone osteosarcoma epithelial

cells, MCF10A - human mammary epithelial cells, rabbit fibroblasts* - rabbit stromal cells from cornea differentiated to fibroblasts, BAEC - bovine aortic endothelial cells, BPASMC - bovine pulmonary artery smooth muscle cells, NG108-15 - mouse neuroblastoma/glioblastoma cells, MDCK - Madin-Darby Canine Kidney epithelial cells, MDA-MB-231 - human breast adenocarcinoma epithelial cells, HESC - human embryonic stem cells, HeLa - human cervix adenocarcinoma cells, hiPSC-CM - cardiomyocytes differentiated from human induced pluripotent stem cells, miPSC-CM - cardiomyocytes differentiated from mouse induced pluripotent stem cells, mES-CM - cardiomyocytes differentiated from mouse embrynoic stem cells, SW480 - human colorectal carcinoma cells, CHO-K1 - Chinese hamster ovary cells, Ost - osteoblasts, MEF - mouse embryonic fibroblasts, REF - rat embryonic fibroblasts, IMR-90 - human fetal lung fibroblasts, RT112 - human urinary bladder carcinoma cells, PC12 - rat adrenal gland cells, RPME - rat pulmonary microvascular endothelial cells, *M. xanthus* - *Myxococcus xanthus* bacteria,*D. discoideum* - *Dictyostelium discoideum*, DRG - rat dorsal root ganglion neurons, RVM - neonatal rat ventricular myocytes

Material abbreviations: PA - polyacrylamide, PDMS - polydimethylsiloxane, PEGDA - polyethylene (glycol) diacrylate

Abbreviations in methods: PIV - particle image velocimetry, PIV-q - particle image velocimetry using quadratic differences, DIC - digital image correlation, CC - cross correlation, WA - water algorithm, PTV - particle tracking velocimetry, SVD - singular value decomposition, FTTC - Fourier transform traction cytometry, unc - unconstrained, con - constrained, BEM - boundary element method, FEM - finite element method, LIBTRC - custom traction mapping software created by Dembo and Wang 1999 [58], TRPF - traction reconstruction with point forces supplied by user [65], ZO - Zero order, LC - L-corner criterion, DT - Discrepancy Theorem,FEA - finite element analysis, TF - traction force, TS - traction stress, TTF - total traction force, FA - focal adhesion
